# Novelty in the development of biodegradable polymer coatings for biomedical devices: paclitaxel grafting on PDMMLA derivatives

**DOI:** 10.1080/15685551.2022.2054116

**Published:** 2022-03-20

**Authors:** Elnaz Gholizadeh, Meriem Naim, Rima Belibel, Hanna Hlawaty, Christel Barbaud

**Affiliations:** a Institut Galilée, Laboratory for Vascular Transitional Science (LVTS), Université Sorbonne Paris Nord, Villetaneuse, France; b Smbh, Laboratory for Vascular Transitional Science (LVTS), Université Sorbonne Paris Nord, Bobigny, France; c Université Sorbonne Paris Nord, KymiaNova, Châtenay Malabry, France

**Keywords:** PDMMLA, polyesters, paclitaxel, covalent grafting, steglich esterification, drug eluting polymers

## Abstract

Biocompatible and biodegradable polymers are widely used in the medical field. In some cases, the biopolymer is accompanied by an active drug, which is delivered locally in a controlled manner in order to improve the healing conditions. Poly([R,S]-3,3-dimethylmalic acid) (PDMMLA) is a synthetic amphiphilic biodegradable polymer, which unlike PLA, can be chemically modified to adapt hydrophilic/hydrophobic balance, degradation kinetics, and physicochemical and biological properties. It may contain a lateral alkyl group or a functional group for coupling bioactive molecules to release during its degradation. In this work, we realized the chemical grafting of paclitaxel (PTX), a microtubule stabilizing anti-cancer agent on PDMMLA derivatives bio-polyesters following a Steglich esterification protocol. 1D and 2D NMR analyses validated the reaction with 10% (using 0.1 equivalent) of PTX on the copolymer PDMMLAH_40_-*co*-Hex_60_ (PDMMLA 40/60) and a maximal PTX grafting rate of 55% on the homopolymer PDMMLAH (PDMMLA 100/0). *In vitro* adhesion and cytotoxicity assays were carried out on HUVEC cells with PDMMLA 40/60, PDMMLA-PTX 30/10/60 and PLA.

## 1. Introduction

Biologically active materials are of great interest in medical fields for the treatment of various diseases. Bioresorbable polymers especially draw attention because of their specific properties. They can be used as suture threads, scaffolds for tissue regeneration and regrowth, or as vectors for local drug delivery. In this case, the polymer is used as a matrix loaded with drugs which is implanted into the area of interest, releasing the drug during the healing period and being degraded naturally at the same time [1,2]. PLA is one of the most used biopolymers in the development of drug-eluting devices, showing interesting biocompatible and bioresorbable properties. However, the slow degradation kinetics of PLA going up to 2 years is considered as a restrictive drawback. In the case of drug-eluting stents (DESs) for example, the durable presence of the polymer coating can lead to a late stent thrombosis [^3–5^].

Here, we study the chemical grafting of paclitaxel (PTX) on new bioresorbable polyesters, PDMMLAs, derived from poly(β-malic acid) (PMLA), which is also a polyester known for its biocompatible and biodegradable properties [6,7]. Previous studies have shown the promising properties of PDMMLA derivatives in terms of mechanical properties [8,9], degradation kinetics [10], and cell response [11]. The most interesting point about PDMMLA is the presence of carboxylic acid groups on its side chain, allowing control of the hydrophilicity levels of the polymer, which affects its degradation kinetics. It also makes possible the functionalization of these groups using potential molecules such as active drugs. It is supposed that when the drug is covalently bonded to the polymer, its release can be slower and controlled compared to the cases where the drug is loaded to the polymer only by physical interactions leading to a burst release [12].

PTX is an FDA approved anti-cancer agent, isolated for the first time from the Pacific yew tree, *Taxus brevifolia*. As a member of the taxane family, PTX acts through binding to β-tubulin and inhibiting the disassembly of microtubules [13]. This microtubule stabilization during the mitotic stage inhibits cell division, resulting in cell cycle blocking and cell death [14,15]. PTX is widely used for the treatment of ovarian, lung, prostate, breast, and solid tumor cancer. In recent years, it is also being used as an anti-proliferative agent in DESs, since it prevents the migration and the proliferation of smooth muscle cells (SMCs), limiting the neointima formation, and thus intra-stent restenosis [16].

The Steglich esterification protocol is followed in this work, in order to form an ester bond between the hydroxyl group of PTX and the carboxylic acid group of PDMMLA. In this protocol, the carboxylic acid is activated by *N,N’*-dicyclohexylcarbodiimide (DCC), forming an *O*-acylisourea intermediate [17,18]. The esterification is improved by the addition of a catalytic amount of 4-(dimethylamino)pyridine (DMAP), leading to a more effective reaction [19,20]. According to the literature, by using DCC/DMAP, only the 2’-hydroxyl of the PTX is activated [21,22].

In order to obtain the desired amphiphilic bioactive polymer, the grafting of PTX was first realized on a PDMMLA copolymer containing 40% of carboxylic acid groups and 60% of hexylic ester groups, PDMMLAH_40_-*co*-Hex_60_ (PDMMLA 40/60). Indeed, previous research studies have shown that the amphiphilic copolymer PDMMLAH_30_-*co*-Hex_70_ (PDMMLA 30/70) is the most optimal surface for grafting the desired therapeutic agent. It presents better adhesion and proliferation of human endothelial cells [11], very favorable surface properties [8,9] and adequate degradation rate in physiological conditions [10].

**Figure 1.**
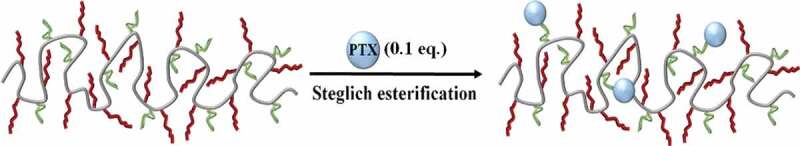
Schematic representation of PTX covalent grafting on PDMMLA 40/60 *via* Steglich esterification; Red: hexylic ester groups, Green: carboxylic acid groups, Blue: PTX.

Relying on these results, a new PDMMLA derivative was prepared with 40% of -COOH (PDMMLA 40/60), and 10% of PTX was grafted on these groups in order to recover the initially desired 30% carboxylic acid groups (Figure 1).

In addition, the hydrophilic homopolymer PDMMLA 100/0, which contains only the repeating unit of -COOH groups on its side chain, is selected in order to determine the maximal grafting percentage of PTX

The grafting is confirmed by 1D and 2D NMR analyses. Thereafter, thermogravimetric analysis (TGA) and differential scanning calorimetry (DSC) are utilized to study the thermal properties of the polymers.

It is also important to test the bioactive terpolymer PDMMLA-PTX 30/10/60 *in vitro* and to compare it with the corresponding copolymer PDMMLA 40/60 and the PLA which is the most studied. For this, cell adhesion tests of these three polymers on HUVEC endothelial cells as well as cytotoxicity tests will be carried out.

## 2. Materials and methods

### 2.1. Materials

All commercially available chemicals were purchased from Sigma Aldrich (France) and employed as received. Reactions with anhydrous organic solvents were performed under nitrogen atmosphere. THF was distilled on sodium-benzophenone.

FTIR spectra were recorded on AVATAR 370 TF-IR Thermo Nicolet spectrometer using the Nicolet OMNI-Sampler ATR Smart Accessory (Ge, DTGS). Adsorption bands are given in cm^−1^.

^1^H and ^13^C and HMBC NMR spectra were recorded on a Bruker AM-400 MHz spectrometer using deuterated chloroform (CDCl_3_) or deuterated acetone (CD_3_COCD_3_) as solvent, depending on the sample solubility. Chemical shifts (δ) are given in ppm. Multiplicity of the groups is given as follows: s (singlet); d (doublet); t (triplet); q (quadruplet); m (multiplet).

The absolute molecular weights and weight distributions were determined at room temperature by coupling a high-performance size-exclusion chromatography (HPSEC), a multi-angle laser light scattering detector (MALLS), a viscosimeter, and a differential refractive index (dRI) detector. THF was used as the carrier phase and was filtered through a 0.1 µm filter unit (Millipore, Billerica, USA). It was degassed (DGU-20A3R Shimadzu, Kyoto Japan) and eluted at a 0.5 mL/min flow rate (LC10Ai Shimadzu, Kyoto Japan). 100 µL of a 0.2 µm-filtered sample solution (C = 10 mg/mL) were injected with an automatic injector (SIL-20A HT Shimadzu, Kyoto Japan). The column packing was a divinylbenzene gel. The MALLS photometer, a miniDawn TREOS from Wyatt Technology Inc. (Santa Barbara, CA, USA) was provided with a fused silica cell and a Ga-As laser (λ = 665.8 nm). The whole collected data: light scattering (LS), dRI were analyzed using the Astra v6.0.6 software package. Molar mass was obtained with a Zimm order 1 method. The concentration of each eluted fraction was determined with dRI (RID10A Shimadzu, Kyoto Japan) with a dn/dc value of 0.05.

Thermogravimetric analysis (TGA) measurements were carried out on a TGA Q50 analyzer. The temperature range was set from 20°C to 500°C with a heating rate of 10°C/min under N_2_ atmosphere. Differential scanning calorimetry (DSC) analyses were carried out on a DSC Q2000 analyzer. Polymers were put in the furnace and heated from −25°C to 200°C with a heating rate of 10°C/min (the final temperature can vary for different polymers regarding their TGA values). The heating cycle was repeated twice, and the *T_g_* value was collected from the inclination point on the second heating curve.

### 2.2. Methods

*Polymer synthesis*: PDMMLAs were prepared in anhydrous THF solution by ring-opening polymerization of racemic β-lactones following the previously reported protocol, using tetraethylammonium benzoate as initiator [23]. β-lactones were also synthesized following the previously described procedure with different functional groups to bring the hydrophilic and hydrophobic properties to the polymer [24].

*PDMMLAH_40_-co-Hex_60_ (PDMMLA 40/60)*: For the synthesis of this copolymer, β-lactones with benzylic and hexylic groups were used. Theoretical molecular weight of the polymer can be determined by the molar ratio of initiator/monomer (I/M). In this work, an initiator was used at (I/M = 0.005) equivalent per mole monomer. 4-benzyloxycarbonyl-3,3-dimethyl-2-oxetanone (benzylic lactone) (1.1 g, 4.67 mmol) and 4-hexyloxycarbonyl-3,3-dimethyl-2-oxetanone (hexylic lactone) (1.6 g, 7.01 mmol) were dissolved in 50 mL of freshly distilled THF. The lactone solution was then added to a round bottom flask containing the initiator tetraethylammonium benzoate (14.65 mg, 0.05 mmol) under nitrogen atmosphere. After confirmation of the polymerization by FTIR, two to four drops of acetic acid were added in order to obtain PDMMLA-Bn_40_-*co*-Hex_60_ (M_n_ = 4.390 × 10^4^ g/mol; M_w_ = 4.391 × 10^4^ g/mol; Ð = 1.00). The formed polymer was isolated by precipitation in ethanol. A hydrogenolysis was then carried out on the polymer using 920 mg (40% of the total mass) of Pd/C for 2.3 g (1 eq.) of PDMMLA-Bn_40_-*co*-Hex_60_. PDMMLAH_40_-*co*-Hex_60_ (PDMMLA 40/60) was thus obtained. *T_d_* = 198.1°C; *T_g_* = 23.66°C. FTIR (neat, cm^−1^) 1743 (C = O ester). **^1^H-NMR (400 MHz, CD_3_COCD_3_,δ_H_ ppm**): 0.69 (s, 3 H, H_l_), 1.22 (m, 12 H, H_f_, H_e_, H_k_, H_j_, H_i_), 1.54 (s, 2 H, H_h_), 4.06 (s, 2 H, H_g_), 5.26 (s, 1H, H_b_). **^13^C-NMR (100 MHz, CD_3_COCD_3_,δ_C_ ppm)**: 13.99 (C_l_), 22.49 (C_f_, C_e_), 26.31 (C_k_), 32.22 (C_i_, C_j_, C_k_), 45.98 (C_c_), 66.28 (C_g_), 77.14 (C_b_), 168.26 (C_a_), 173.76 (C_d_).

*PDMMLAH (PDMMLA 100/0)*: The same protocol was followed for the synthesis of the homopolymer using the benzylic β-lactone (1.01 g, 4.27 mmol, 1 eq.) and the initiator tetraethylammonium benzoate (5.35 mg, 0.021 mmol, 0.005 eq.) (M_n_ = 3.180 × 10^4^ g/mol; M_w_ = 3.180 × 10^4^ g/mol; Ð = 1.00). PDMMLA-H (PDMMLA 100/0) was obtained after a catalytic hydrogenolysis using 500 mg of Pd/C (1 mass eq.) for 500 mg (1 eq.) of PDMMLA-Bn. *T_g_* = 69.9°C. FTIR (neat, cm^−1^) 1746 (C = O ester). **^1^H-NMR (400 MHz, CD_3_COCD_3_,δ_H_ ppm)**: 1.26 (m, 6 H, H_e_, H_f_), 5.24 (s, 1H, H_b_). **^13^C-NMR (100 MHz, CD_3_COCD_3_,δ_C_ ppm)**: 20.64 (C_e_, C_f_), 44.91 (C_c_), 76.04 (C_b_), 168.34 (C_a_), 173.14 (C_d_).

*PDMMLAH_30_-ter-PTX_10_-ter-Hex_60_*: Copolymer PDMMLA 40/60 (600 mg, 2.59 mmol, 1 eq.), PTX (221.1 mg, 0.25 mmol, 0.1 eq.), and DMAP (87.3 mg, 0.71 mmol, 10% of total mass) were placed in a round-bottom flask and dissolved in 20 mL of freshly distilled THF under nitrogen atmosphere. *N,N’*-Dicyclohexylcarbodiimide (DCC) (53.4 mg, 0.25 mmol, 0.1 eq.) was dissolved in 5 mL of THF as well and added to the previous mixture using a cannula. The reaction was stirred under nitrogen atmosphere for 48 h at room temperature. After the reaction, the polymer was isolated by precipitation in cyclohexane. M_n_ = 3.178 × 10^4^ g/mol, M_w_ = 3.185 × 10^4^ g/mol, Ð = 1.002. *T_d_* = 168.86°C; *T_g_* = 34.70°C. **^1^H-NMR (400 MHz, CDCl_3_,δ_H_ ppm**): 0.87 (s, 3 H, H_l_), 1.24 (m, 18.05 H, H_16_, H_18_, H_e_, H_f_, H_k_, H_j_, H_i_), 1.61 (s, 2.05 H, H_h_), 1.67 (s, 0.3 H, H_17_), 1.79 (s, 0.29 H, H_19_), 1.86, 2.51 (td, 0.27 H, H_6a_, H_6b_), 2.23 (s, 0.34 H, H_31_), 2.26 (s, 0.3 H, 1-OH), 2.32, 2.38 (m, 0.33 H, H_14a_, H_14b_), 2.41 (s, 0.3 H, H_29_), 2.49 (d, 0.29 H, 7-OH), 3.77 (m, 0.11 H, H_3_), 4.12 (m, 2.13 H, H_g_, H_20a_), 4.28 (d, 0.12 H, H_20b_), 4.39 (m, 0.11 H, H_7_), 4.80 (m, 0.12 H, H_2’_), 4.95 (dd, 0.12 H, H_5_), 5.34 (s, 1.32 H, H_b_), 5.66 (d, 0.12 H, H_2_), 5.78 (d, 0.12 H, H_3’_) 6.20 (t, 0.12 H, H_13_), 6.27 (s, 0.12 H, H_10_), 7.00 (d, 0.10 H, N-H), 7.28 (m, 0.13 H, H_35_), 7.31 (m, 0.22 H, H_34_, H_36_), 7.42 (m, 0.21 H, H_40_, H_42_), 7.48 (m, 0.11 H, H_41_), 7.51 (m, 0.24 H, H_33_, H_37_), 7.52 (d, 0.24 H, H_24_, H_26_), 7.64 (t, 0.12 H, H_25_), 7.76 (d, 0.23 H, H_39_, H_43_), 8.15 (d, 0.21 H, H_23_, H_27_). **^13^C-NMR (100 MHz, CDCl_3_,δ_C_ ppm)**: 9.58 (C_19_), 13.98 (C_l_), 14.83 (C_18_), 20.87 (C_31_), 21.82 (C_16_), 22.48 (C_e_, C_f_), 22.60 (C_29_), 25.39 (C_j_) 26.80 (C_17_), 28.38 (C_k_), 29.69 (C_i_), 30.31 (C_h_), 35.60 (C_14_), 35.62 (C_6_), 43.15 (C_15_), 45.18 (C_c_), 45.64 (C_3_), 55.25 (C_3’_), 58.51 (C_8_), 65.86 (C_g_), 72.15 (C_7_), 73.29 (C_2’_), 74.76 (C_2_), 74.91 (C_13_), 75.62 (C_10_), 76.50 (C_b_), 77.29 (C_20_), 78.93 (C_1_), 81.06 (C_4_), 84.43 (C_5_), 127.07 (C_39_, C_43_), 127.17 (C_33,_ C_37_), 128.29 (C_35_), 128.68 (C_40,_ C_42_), 128.74 (C_24,_ C_26_), 129.96 (C_34,_ C_36_), 129.14 (C_22_), 130.20 (C_23,_ C_27_), 131.99 (C_41_), 133.06 (C_11_), 133.55 (C_32_), 133.74 (C_25_), 137.92 (C_38_), 142.07 (C_12_), 166.94 (C_5’_), 167.47 (C_21_), 167.70 (C_a_), 170.50 (C_28_), 171.31 (C_30_), 172.74 (C_1’_), 173.15 (C_d_), 203.65 (C_9_).

*PDMMLAH_45_-co-PTX_55_*: Following the same protocol, PTX was grafted to the homopolymer PDMMLA 100/0 using the following quantities of reactants: PDMMLA100/0 (16.8 mg, 0.12 mmol, 1 eq.), PTX (100.0 mg, 0.12 mmol, 1 eq.), DMAP (14.1 mg, 0.11 mmol, 10% of total mass), DCC (24.1 mg, 0.12 mmol, 1 eq.) and 20 mL of anhydrous THF. M_n_ = 2.883 × 10^4^ g/mol, M_w_ = 2.974 × 10^4^ g/mol, Ð = 1.031. *T_d_* = 170.1°C; *T_g_* = 64.53°C. **^1^H-NMR (400 MHz, CDCl_3_,δ_H_ ppm**): 1.15 (m, 3.00 H, H_16_) 1.20 (m, 9.22 H, H_17_, H_f_, H_e_), 1.57 (s, 2.99 H, H_18_), 1.68 (s, 3.04 H, H_19_), 1.77, 2.41 (td, 2 H, H_6a_, H_6b_), 2.19 (s, 3.06 H, H_31_), 2.18 (s, 1. 03 H, 1-OH), 2.32, 2.38 (m, 2 H, H_14a,_ H_14b_), 2.40 (s, 3.06 H, H_29_), 3.71 (m, 1.03 H, H_3_), 4.10 (d, 1.02 H, H_20a_), 4.20 (d, 1.01 H, H_20b_), 4.30 (m, 1.03 H, H_7_), 4.70 (s, 1.03 H, H_2’_), 4.80 (dd, 1.06 H, H_5_), 5.25 (s, 0.69 H, H_b_), 5.59 (d, 1.09 H, H_2_), 5.73 (d, 1.06 H, H_3’_), 6.15 (t, 1.00 H, H_13_), 6.23 (s, 1.01 H, H_10_), 7.12 (d, 1.04 H, N-H), 7.27 (m, 1H, H_35_), 7.31 (m, 2 H, H_34_, H_36_), 7.42 (m, 2 H, H_40_, H_42_), 7.48 (m, 1H, H_41_), 7.51 (m, 2 H, H_33_, H_37_), 7.52 (d, 2 H, H_24_, H_26_), 7.64 (t, 1H, H_25_), 7.76 (d, 2 H, H_39_, H_43_), 8.15 (d, 2 H, H_23_, H_27_). **^13^C-NMR (100 MHz, CDCl_3_,δ_C_ ppm**): 9.60 (C_19_), 14.80 (C_18_), 20.85 (C_31_), 21.87 (C_16_), 22.51 (C_e_, C_f_), 22.61 (C_29_), 26.77 (C_17_), 35.58 (C_14_), 35.63 (C_6_), 43.15 (C_15_), 45.16 (C_c_), 45.66 (C_3_), 55.23 (C_3’_), 58.54 (C_8_), 72.17 (C_7_), 73.31 (C_2’_), 74.77 (C_2_), 74.94 (C_13_), 75.66 (C_10_), 76.48 (C_b_), 77.30 (C_20_), 78.91 (C_1_), 82.00 (C_4_), 84.44 (C_5_), 127.10 (C_39_, C_43_), 127.13 (C_33_, C_37_), 128.32 (C_35_), 128.72 (C_40_, C_42_), 128.77 (C_24,_ C_26_), 129.16 (C_22_), 129.93 (C_34_, C_36_), 130.19 (C_23,_ C_27_), 132.03 (C_41_), 133.06 (C_11_), 133.53 (C_32_), 133.77 (C_25_), 137.98 (C_38_), 142.10 (C_12_), 166.91 (C_5’_), 167.44 (C_21_), 167.73 (C_a_), 170.51 (C_28_), 171.29 (C_30_), 172.77 (C_1’_), 173.20 (C_d_), 203.66 (C_9_).

*Cell culture*: HUVECs (Human Umbilical Vein Endothelial cells, N° CRL- 1730, ATCC, LGC Molsheim, France) were cultured in complete medium containing endothelial cell basal medium 2 (ECBM2, PromoCell, Germany) and epidermal growth factor (EGF), hydrocortisone, vascular endothelial growth factor (VEGF), basic fibroblast growth factor (bFGF), insulin-like growth factor, ascorbic acid, heparin, and antibiotics (penicillin-streptomycin 1%, PAA Laboratories, Pasching, Austria). This medium was supplemented with 10% of fœtal bovine serum. Cells were incubated at 37°C in 5% CO_2_ for 24 h to perform the adhesion and cytotoxicity tests.

*Lactate dehydrogenase (LDH) colorimetric assay for cytotoxicity measurement*: 25,000 HUVECs were cultured on different polymer films (PLA, PDMMLA 40/60 or PDMMLA-PTX 30/10/60) with complete culture media. After 24 h, 48 h, and 72 h of cell culture, the cytotoxicity was determined using colorimetric LDH cytotoxicity assay following the manufacturer’s instructions (LDH, CyQUANT™, Invitrogen France) using spectrophotometer at 490 nm. The experiment was produced four times at different cell passage numbers (n = 4).

*Adherence assay*: 3000 HUVECs were plated on Labtek® (Thermo Fisher Scientific, Brébières, France) coated with fibronectin (control) or polymer films (PLA, PDMMLA 40/60 or PDMMLA-PTX 30/10/60) with complete culture media. After 15 minutes of incubation, the cell culture media was removed and the cells were washed twice with PBS. Nuclei were stained with DAPI (dilution 1/1000, Invitrogen, France) and the cytoskeleton (F-actin) was labelled with Alexa fluor 546 phalloidin (dilution 1/200, Invitrogen, France). A fluorescence microscope and Archimed® software were used, x40 magnification to analyze cell adhesion. The experiment was produced three times at different cell passage numbers (n = 3).

*Statistical analysis*: Results are expressed by mean ± SEM. Data were analyzed by ANOVA test *p* < 0.05 represent statistically significant difference.

## 3. Results and discussion

### Preparation of polymer supports for grafting

**Figure 2.**
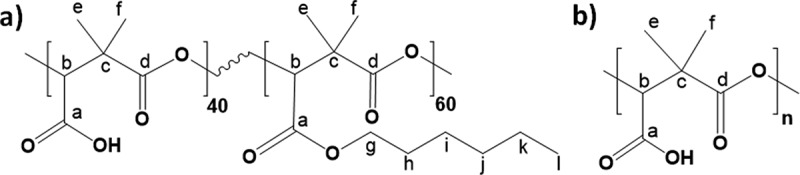
Chemical structure of: a) PDMMLA 40/60, b) PDMMLA 100/0.

Two polymers were utilized as support to carry out the covalent grafting reaction: copolymer PDMMLA 40/60 and homopolymer PDMMLA 100/0 (Figure 2).

**Scheme 1:**
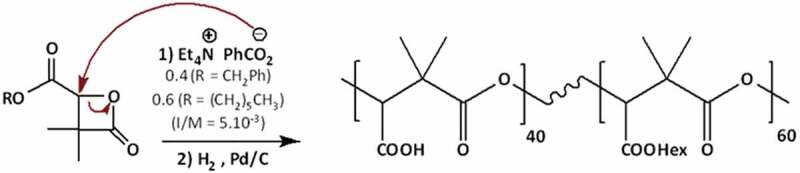
Synthesis of PDMMLA40/60 via the ROP of benzylic and hexylic β-lactones.

These polymers were successfully prepared *via* an anionic ring-opening polymerization (ROP) of the monomers, α,α,β-tri-substituted β-lactones: benzylic β-lactone ‘R = -CH_2_Ph’ and hexylic β-lactone ‘R = -(CH_2_)_5_-CH_3_’ in anhydrous THF solution following the previously reported procedure [23,25]. Tetraethylammonium benzoate was used as initiator (Scheme 1). The polymerization was monitored by FTIR spectroscopy, which shows the disappearance of the β-lactone band (n_C=O_) at 1850 cm^−1^ (100% conversion).

The chemical structure of the polymers was confirmed by ^1^H and ^13^C NMR. The co-monomers relative content (40%) is obtained by ^1^H NMR using the integration ratio of peak at 4.15 ppm corresponding to -CH_2_-O-hexyl and at 5.20 ppm corresponding to -CH_2_-O-benzyl. The final polymers with -COOH groups were obtained after a catalytic hydrogenolysis using palladium on charcoal (Scheme 1), confirmed by the disappearance of benzylic peaks at 5.2 and 7.4 ppm on the ^1^H-NMR spectrum for both PDMMLA 40/60 and PDMMLA 100/0.

### Covalent grafting

**Scheme 2:**
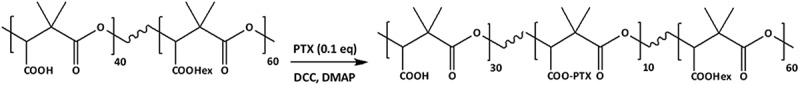
Covalent grafting of 10% of PTX on the PDMMLA 40/60.

The approach was to synthesize a bioresorbable polymer with a covalently grafted active drug. The reaction was realized *via* the Steglich esterification protocol, which forms a new ester bond between the 2’-OH group of PTX and the -COOH groups of the polymers (Scheme 2).

**Figure 3.**
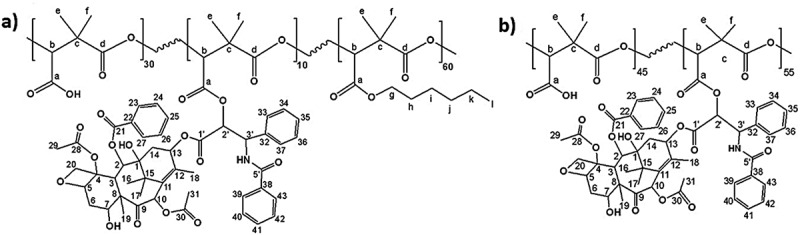
Chemical structure of (a) PDMMLA-PTX 30/10/60 and (b) PDMMLA-PTX 45/55.

Final polymers obtained after PTX grafting on PDMMLA 40/60 and PDMMLA 100/0, are the terpolymer PDMMLAH_30_-*ter*-PTX_10_-*ter*-Hex_60_ (PDMMLA-PTX 30/10/60) and the copolymer PDMMLAH_45_-*co*-PTX_55_ (PDMMLA-PTX 45/55), respectively (Figure 3). The new terpolymer PDMMLA-PTX 30/10/60 is particularly important since its development aims biomedical applications.

**Figure 4.**
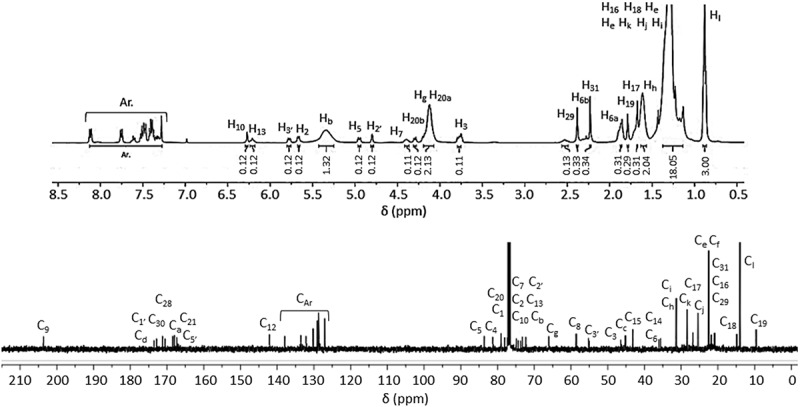
^1^H (400 MHz) and ^13^C (100 MHz)(CDCl_3_) NMR spectra of the new terpolymer PDMMLA-PTX 30/10/60.

**Figure 5.**
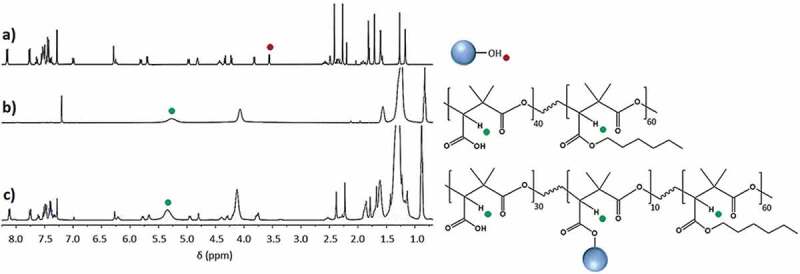
^1^H-NMR (400 MHz, CDCl_3_) spectra of (a) PTX (represented by the blue round shape with its available 2’-hydroxyl group), (b) PDMMLA 40/60, and (c) PDMMLA-PTX 30/10/60.

Figure 4 shows the ^1^H and ^13^C-NMR spectra of the terpolymer PDMMLA-PTX 30/10/60 with the assignments of the peaks, and Figure 5 shows the comparative ^1^H-NMR spectra of PTX, PDMMLA 40/60 and PDMMLA-PTX 30/10/60. Chemical shifts and integrals show that the grafting by esterification has taken place involving the 2’-hydroxyl group of PTX and the carboxylic acid group of the copolymer. The peak at 3.55 ppm on the spectrum ‘a’ corresponding to the proton of the alcohol on C_2ʹ_of PTX (Figure 5 –a) disappears on the spectrum ‘c’ (Figure 5–c) (after grafting), indicating that this functional group has been transformed into an ester. Using the integral values of the peak at 5.34 ppm (integrating for 1.32, proton b on the polymer chain) and the peak at 6.20 ppm (integrating for 0.12, proton H_2’_), one can deduce the ratio of the PTX-ester groups and the remaining carboxylic acid groups on the side chain of the polymer. Calculations confirm that 10% of PTX has been grafted on PDMMLA 40/60. The desired proportion of PTX and the new terpolymer are thus successfully obtained: 10% of PTX, terpolymer PDMMLA-PTX 30/10/60 (Figure 3–a).

**Figure 6.**
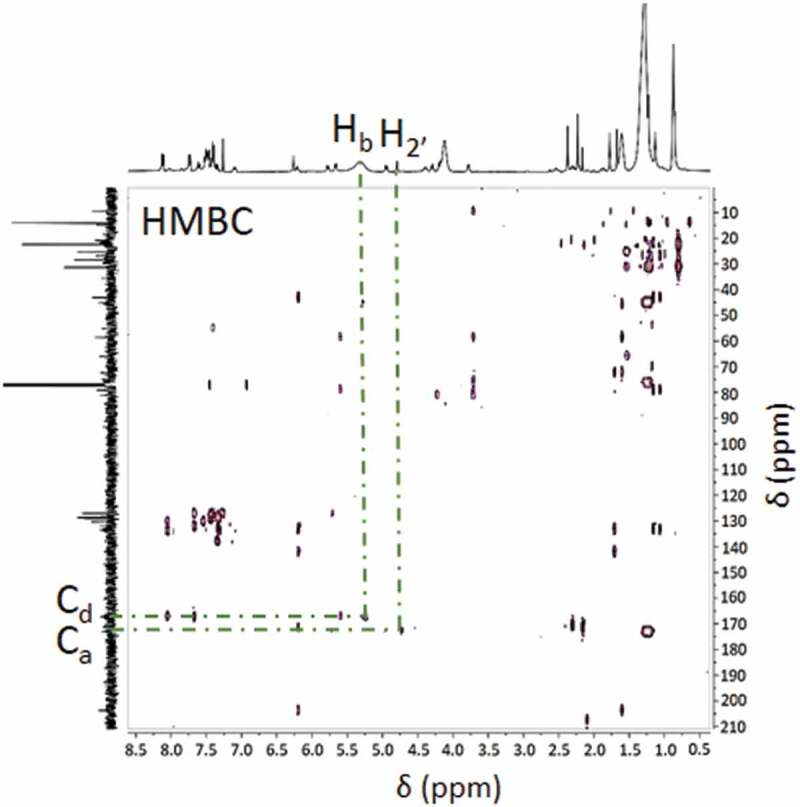
HMBC spectrum of the terpolymer PDMMLA-PTX 30/10/60 in CDCl_3._

In addition to ^1^H and ^13^C spectra, a heteronuclear multiple-bond correlation (HMBC) of the PDMMLA-PTX 30/10/60 was recorded in order to demonstrate the formation of a new bond (Figure 6). An HMBC spectrum represents the correlations between two different nuclei (here, carbons, and protons) through several bonds [26]. In our case, the terpolymer shows a correlation between H_2’_ and C_a_, which are through three bonds (*^3^J*) and a correlation between H_b_ and C_d_ (*^3^J*). This method confirms with evidence the formation of the ester bond by demonstrating the correlation between the groups directly involved in this new bond, H_2’_ and C_a_ (*^3^J*).

**Figure 7.**
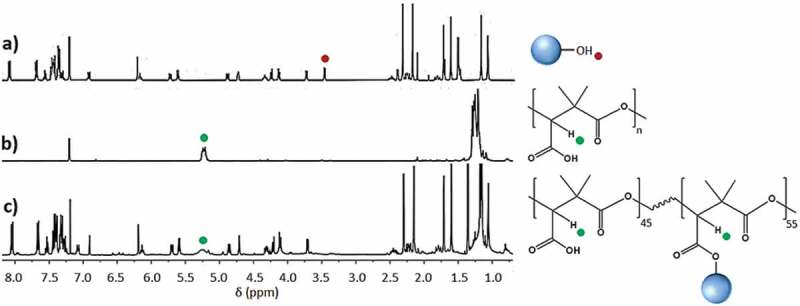
^1^H-NMR (400 MHz, CDCl_3_). spectra of: a) PTX, b) PDMMLA-H, c) PDMMLA-PTX 45/55.

Regarding the homopolymer, NMR signals (Figure 7) of the same functional moieties show that there is 45% of free carboxylic acid groups on the PTX-grafted PDMMLA, compared to the PDMMLA 100/0. This indicates that the maximal grafting percentage is limited to around 55%, which can be explained by the relatively large structure of PTX and its consequent steric hindrance. The obtained copolymer is thus the PDMMLAH_45_-*co*-PTX_55_ or PDMMLA-PTX 45/55 (Figure 3–b).

### Thermal properties

TGA and DSC analyses were performed in order to determine the thermal properties of the polymers. The TGA measurements provide information about the thermal stability and the degradation temperature *T_d_* of the polymers [27]. The DSC technique determines mainly the glass transition temperature *T_g_* and melting temperature *T_m_* of the polymer [28,29].

**Figure 8.**
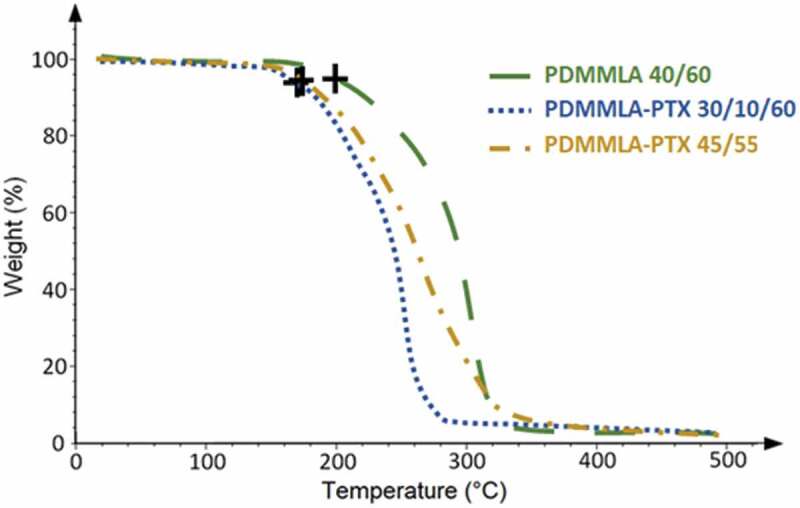
TGA thermograms of PDMMLA 40/60, PDMMLA-PTX 30/10/60, and PDMMLA-PTX 45/55.

Figure 8 presents the TGA thermograms of PDMMLA 40/60, PDMMLA-PTX 30/10/60, and PDMMLA-PTX 45/55. The onset of thermal degradation of each of the polymers can be observed between 170°C and 200°C. This degradation is reflected by a loss of the residual mass of the sample. In our case, the complete degradation of the polymers is observed between 270°C and 320°C.

**Figure 9.**
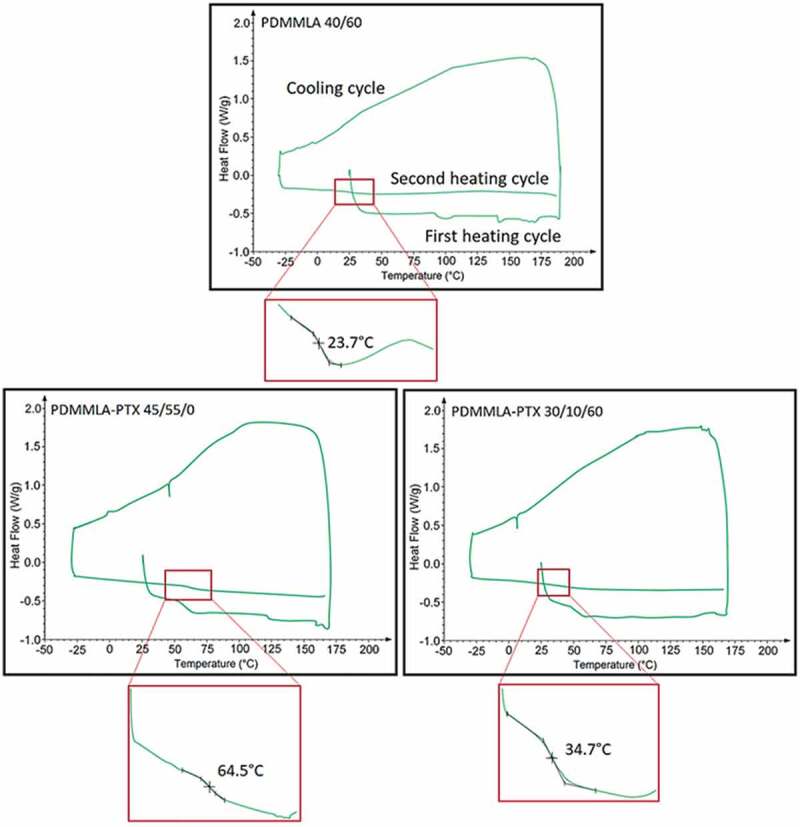
DSC thermograms of PDMMLA 40/60, PDMMLA-PTX 30/10/60, and PDMMLA-PTX 45/55. T_g_ is measured during the second heating cycle.

The DSC curves of the polymers are shown in Figure 9. Results show that the grafting of 10% of PTX on the copolymer PDMMLA 40/60 increases the *T_g_* from 23.7°C to 34.7°C. In contrast, in the case of the homopolymer PDMMLA-H, the *T_g_* undergoes a slight decrease from 69.9°C to 64.5°C [11]. On the other hand, a *T_g_* value of 34.7°C is obtained for the terpolymer PDMMLA-PTX 30/10/60. A *T_g_* below 37°C indicates that the polymer will be in its rubbery state at physiological temperature and consequently, will not undergo a change after implantation in the body.

### Cell response

**Figure 10.**
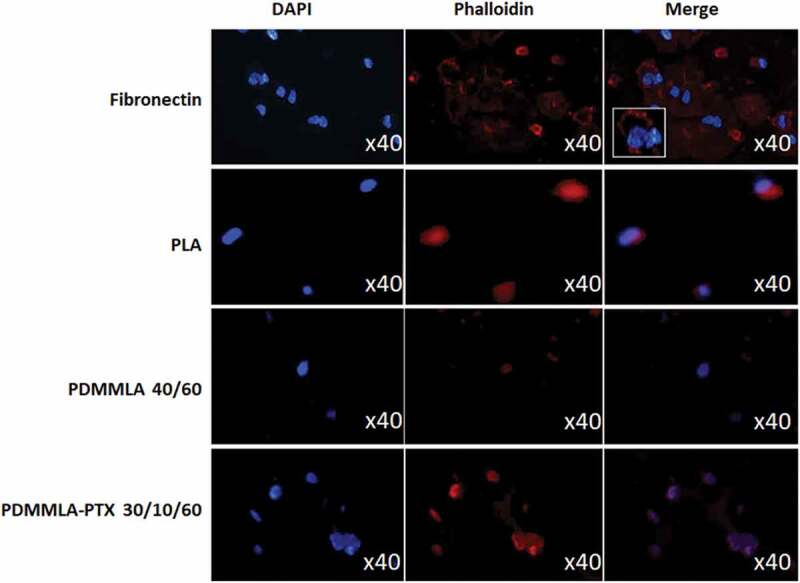
Adhesion assay results on control (fibronectin), PLA, PDMMLA 40/60, and PDMMLA-PTX 30/10/60. For adhesion assay, HUVECs were seeded in Labtek™ coated with fibronectin or polymers films (PLA, PDMMLA 40/60 or PDMMLA-PTX 30/10/60) and incubated for 15 minutes in complete culture media. Then, DAPI staining (nucleus, first column) and Phalloidin staining (cytoskeleton, second column) were carried out. The merge images of the two stainings are presented in the third column. The images were taken by fluorescence microscopy (x40 magnification, high view insert in merge). Three independent experiments were carried out (n = 3).

*Adhesion*: Cell adhesion assay was performed *in vitro* to study the attachment of HUVECs on different polymer layers (PLA, PDMMLA 40/60 or PDMMLA-PTX 30/10/60) and compared to fibronectin. Consequently, after 15 minutes of HUVEC incubation, we noticed an important attachment of HUVECs on PDMMLA-PTX 30/10/60 layer, which was similar to cells attached on fibronectin. In contrast, less of HUVECs were attached on PLA and PDMMLA 40/60 (Figure 10).

**Figure 11.**
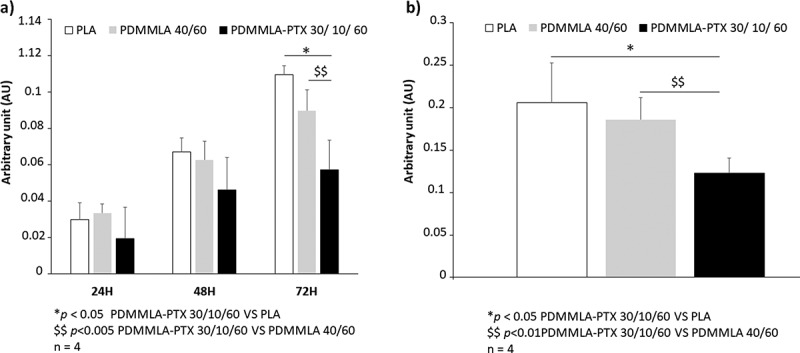
Cytotoxicity results on PLA, PDMMLA 40/60, and PDMMLA-PTX 30/10/60. For cytotoxicity assay, HUVECs were plated on Labtek™ coated with PLA, PDMMLA 40/60 or PDMMLA-PTX 10/30/60. Figure 11a: LDH released in cell culture supernatant is proportional to cytotoxicity and was measured by colorimetric assay, as function of HUVECs incubation times (24 h, 48 h and 72 h), Figure 11b: the results of the three times of incubation were summed up to assess the global cytotoxicity of each polymer. Four independent experiments were performed. *p < 0.05 PDMMLA-PTX 30/10/60 vs PLA; $$ p < 0.005 PDMMLA-PTX 30/10/60 vs PDMMLA 40/60; n = 4.

*Cytotoxicity* in HUVEC seeded on the different polymers (PLA, PDMMLA 40/60, and PDMMLA-PTX 30/10/60) was analyzed using LDH assay after 24 h, 48 h, and 72 h of incubation in complete culture media. The level of LDH was analyzed in conditioned media and was proportional to cell apoptosis. Our results showed that PLA and PDMMLA 40/60 induced more cytotoxicity as compared to PDMMLA-PTX 30/10/60 without any significant difference at 24 h and 48 h of cell culture. However, there was a significantly lower cytotoxicity in HUVECs cultured on PDMMLA-PTX 30/10/60 (0.05 ± 0.004) as compared to PLA (0.011 ± 0.013) and to PDMMLA 40/60 (0.09 ± 0.008) at 72 h of cell culture. In parallel, the cytotoxicity analyses during 3 days was gathered (24 h, 48 h and 72 h) and the result showed a significant decrease in cytotoxicity in HUVECs cultured on PDMMLA-PTX 30/10/60 (0.123 ± 0.014) as compared to PLA (0.206 ± 0.03) and PDMMLA 40/60 (0.186 ± 0.019) (Figures 11a and 11b).

## 4. Conclusion

The tailored synthesis of PDMMLA derivatives allows to obtain amphiphilic copolymers with hexylic ester (-COOHex) and carboxylic acid (-COOH) side groups. The presence of chemically modifiable -COOH groups makes possible a covalent grafting of molecules on PDMMLA, such as active drugs. The covalent grafting would improve the release profile and kinetics of the active drug during the healing period.

In this work, the chemical grafting of PTX, an FDA approved active drug, was carried out on two PDMMLA derivatives following the Steglich esterification protocol. The grafting was implemented on the copolymer PDMMLA 40/60 using 0.1 equivalent of PTX per repetition unit, allowing to obtain the terpolymer PDMMLA-PTX 30/10/60, which is the product of interest for future biomedical applications, such as cardiovascular stent coating. 1D and 2D NMR analyses confirm the reaction and the formation of a new bond between PTX and the copolymer.

Following the same protocol, the grafting was also carried out on the homopolymer PDMMLA-H, using 1 equivalent of PTX in order to determine the maximal grafting percentage. NMR analyses of the products show that the grafting of PTX on PDMMLA-H is limited to 55% due to its steric hindrance.

Thermogravimetric analyses of the polymers before and after PTX grafting gave high *T_d_* values for all polymers and a *T_g_* value below 37°C for the terpolymer PDMMLA-PTX 30/10/60 (34.7°C), which is suitable for biomedical applications.

For the cell adhesion test, we observed the more efficient binding of the HUVECs on the PDMMLA-PTX 30/10/60 layer. Cytotoxicity analyses for 3 days (24 h, 48 h, and 72 h) showed the lowest cytotoxicity for HUVECs cultured on PDMMLA-PTX 30/10/60.

All these positive results lead to the conclusion that the PTX grafting reaction has made it possible to develop a new biodegradable and non-cytotoxic terpolymer to be used as a future coating for endovascular stents.
